# Shape Anisotropic Iron Oxide-Based Magnetic Nanoparticles: Synthesis and Biomedical Applications

**DOI:** 10.3390/ijms21072455

**Published:** 2020-04-01

**Authors:** Raquel G. D. Andrade, Sérgio R. S. Veloso, Elisabete M. S. Castanheira

**Affiliations:** Centre of Physics (CFUM), University of Minho, Campus de Gualtar, 4710-057 Braga, Portugal; raquel.gau@gmail.com (R.G.D.A.); sergioveloso96@gmail.com (S.R.S.V.)

**Keywords:** anisotropy, magnetic nanoparticles, hyperthermia, magnetic resonance imaging, drug delivery

## Abstract

Research on iron oxide-based magnetic nanoparticles and their clinical use has been, so far, mainly focused on the spherical shape. However, efforts have been made to develop synthetic routes that produce different anisotropic shapes not only in magnetite nanoparticles, but also in other ferrites, as their magnetic behavior and biological activity can be improved by controlling the shape. Ferrite nanoparticles show several properties that arise from finite-size and surface effects, like high magnetization and superparamagnetism, which make them interesting for use in nanomedicine. Herein, we show recent developments on the synthesis of anisotropic ferrite nanoparticles and the importance of shape-dependent properties for biomedical applications, such as magnetic drug delivery, magnetic hyperthermia and magnetic resonance imaging. A brief discussion on toxicity of iron oxide nanoparticles is also included.

## 1. Introduction

Many efforts are being made to overcome the flaws of cancer treatment and diagnosis. Nanomedicine is the field of nanotechnology responsible for the development of biomedical tools capable of a higher performance than conventional medicine [1,2]. Ferrite nanoparticles are magnetic nanoparticles that show several properties that result from finite-size and surface effects such as high magnetization, superparamagnetism and extra anisotropy contributions. The fact that these nanoparticles do not retain any magnetization upon removal of an applied magnetic field, along with the low toxicity, biocompatibility and strong magnetic properties, endow iron-based nanoparticles as suitable materials for use in bioimaging, cancer theranostics and drug delivery [3]. The dependence between magnetic behavior and size results from the structure of their magnetic domains. When size decreases to a threshold value (commonly 20 nm [4]), the ferrimagnetic material becomes a single domain which is characterized by a uniform magnetization [5,6]. Spinel ferrite nanoparticles have ferrimagnetic behavior, and this means that the material is composed of magnetic domains, each one composed of antiparallel magnetic moments with different magnitudes, resulting in a net spontaneous magnetic moment. When a magnetic field is applied, all domains have their magnetic moments aligned with the magnetic field, resulting in a large net magnetic moment [5,6,7,8].

Ferrite nanoparticles are composed of different atoms occupying two different lattice sites. Magnetite (Fe_3_O_4_) is a ferrite, but other ferrites can be formed when a Fe^2+^ ion is replaced by another cation (Mn^2+^, Zn^2+^, Co^2+^, Mg^2+^, Ni^2+^) [7,8]. The general structure of a normal spinel structure can be written as (Me^2+^)[Fe^3+^_2_]O_4_, where Me^+2^ refers to divalent cations, occupying tetrahedral positions, and Fe^3+^ serving as trivalent cations, occupying octahedral positions [7,8]. Given their unique structural and magnetic properties, ferrite nanoparticles have been widely used for magnetic drug delivery [9,10,11], magnetic hyperthermia [12,13,14] and magnetic resonance imaging [15,16,17]. Research on preparation methods and characterization of different anisotropic shapes of these nanoparticles is important and it is recently increasing, since desired physicochemical properties and applications can be achieved by simply change structural parameters [18,19,20,21,22]. The magnetic behavior of ferrite nanoparticles is highly influenced by shape anisotropy. For instance, magnetization of an elongated nanoparticles will be easier along their long axis than the short axis [23].

In this review, we aim to explore the recently developed synthetic routes of preparation of different anisotropic nanoparticles, namely elongated nanoparticles, nanofilms, sheets and plates, nanocubes and nanoflowers. Herein, we highlight the importance of utilization of anisotropic iron oxide nanoparticles as alternative materials for an enhanced performance in biomedical applications, such as magnetic drug delivery, magnetic hyperthermia and magnetic resonance imaging (MRI). Also, the influence of size, shape and administered dose on the toxicity of iron oxide nanoparticles is briefly discussed.

## 2. Synthesis of Shape Anisotropic Nanoparticles

Synthesis of nanoparticles with controlled shape has been a main challenge in the last decade [24], yet most of the described methods and strategies are confined to magnetite nanoparticles. A gap is thus kept between the shapes that have been already developed and the metal composition that would be of interest to afford improved magnetic and contrast properties, besides other technological applications.

High-quality nanoparticles are attained by different methods, which can be separated in three main classes: physical, chemical and biological [25]. Physical methods include pulsed laser ablation and pyrolysis, while chemical encompass a wide variety of methods, such as co precipitation, hydrothermal and solvothermal synthesis, thermal decomposition, sol-gel synthesis, sonochemical decomposition, microemulsion, microwave-assisted and electrochemical synthesis [24,25,26]. Biological routes include the bacterial and microorganism synthesis [24,25,26]. The advantages and disadvantages of the common methods are included in Table 1, besides examples of spherical and anisotropic shape nanoparticles obtained through each method.

The crystal shape can be thermodynamically or kinetically controlled. A thermodynamic process is associated with the chemical potential of the reaction, that is related to parameters such as temperature and supersaturation of the solution, while a kinetic process happens when stable nucleation sites in supersaturated regions are present, which reduce the reaction energy barrier [55]. During the thermodynamically-driven growth, the reaction proceeds to reduce the particles surface free energy [23,55]. Here, the two mechanisms, reaction-limited and diffusion-limited, affect differently the rate of deposition of atoms in the nuclei surface. At high solution concentration, in the diffusion-limited regime, monomers are precipitated once on the surface of the NPs, which favours monodisperse nanoparticles, while at low concentration, in the reaction-limited regime, the surface reaction limits the growth and leads to different final shapes [23]. Therefore, parameters such as temperature, pH, solvent and concentration of the precursors affect the final shape [55,56]. Considering the relative surface energies of the crystal facets, different strategies have been studied to favour anisotropic shapes, including the use of shaping ligands, template-assisted synthesis and electrodeposition, and magnetic assembly of nanoparticles [23].

The use of shaping ligands is a common method to obtain anisotropic nanoparticles, which can be induced through: (I) steric inhibition of growth and modification of chemical reactivity of specific crystal planes by chelation of the ligand to specific planes; (II) coalescence of reactants or nuclei and consequent anisotropic growth in templated ligand assembly. Common ligands include surfactants such as oleylamine, dodecyldimethylammonium bromide, cetyltrimethylammonium bromide or chloride, octadecylamine and oleic acid [23,55,56]. Common polymers include polyethyleneimine (PEI), poly(ethylene glycol) (PEG), poly(vinylpyrrolidone) (PVP), poly(acrylic acid) (PAA) and poly(vinyl alcohol) (PVA) [23,55,57]. Small molecules containing halides, small polymers and solvents are also used for coordination of specific crystal planes [55,58]. Here, the functional groups have to be considered as they affect the size, shape and magnetic properties of the crystals [23,58]. Li and co-workers [58] demonstrated that the higher polarity of 3-aminopropanol compared to urea led to an increased confinement effect of the magnetite nanocrystal growth through hydrothermal synthesis, while urea was adequate to control nanoparticle shape owing for its surface plane preference σ{111} > σ{001} > σ{101}. Another example of the capping agent effect was studied by Fatima and co-workers in hydrothermal synthesis of magnetite [59], where cubes and octahedra of magnetite could be obtained using ferrous sulphate heptahydrate in ethylene glycol, and potassium hydroxide that was found to favour a faster growth rate of {111} plane than the {100} plane.

Furthermore, π-acceptor ligands (e.g. CO) are described to decrease saturation magnetization, M_s_, values, while the σ-donors (e.g. NH_2_) do not affect the magnetic properties [23]. Also, post-synthesis surface-functionalization can affect phase distribution, as demonstrated by Daniel and co-workers [60]. The replacement of oleic acid by catechol surface ligands led to the separation into two distinctive magnetic entities, due to reduction of a nanoparticle surface fraction to magnetite, which reduced the anisotropy constant and, consequently, the effective magnetization [60].

### 2.1. Elongated Nanoparticles

Elongated magnetic nanoparticles, mainly nanorods, have been of interest in various industrial [61,62], and biomedical applications [63,64,65]. Classification is based on the external energy required for inversion of the magnetization [61]. Nanorods encompass structures with some nanometers of diameter and length up to 100 nm, while the length of nanowires is larger, and nanotubes are hollow nanorods [23]. The magnetic easy axis and magnetization directed along the long axis, which are determined by the shape anisotropy, lead to an anisotropic response to an external magnetic field: a S-like hysteresis loop is obtained when a magnetic field is applied parallel to the short axis, while along the long axis the hysteresis loop is square-like [23]. The reader is directed to articles [66,67,68,69] for more information on the magnetic behavior of elongated nanoparticles. Different strategies have been developed in recent years for the synthesis of elongated iron oxide nanoparticles of different chemical composition, which are summarized in Table 2.

**Figure 1:**
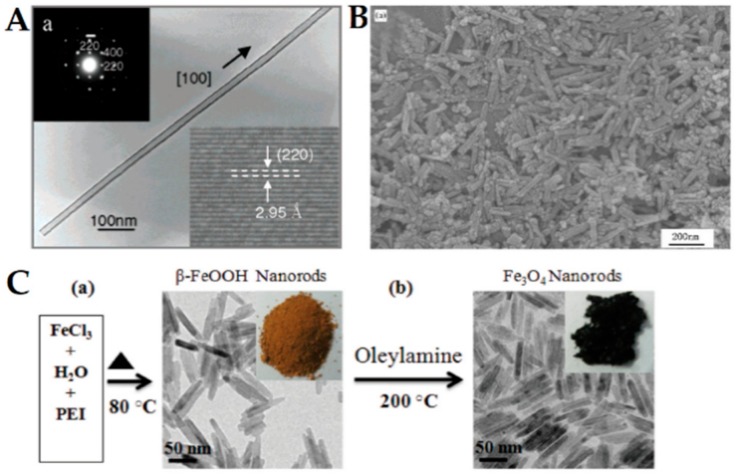
(**A**) TEM image of a Fe_3_O_4_ nanotube. Upper inset: Selected area electron diffraction of the nanotube. Lower inset: HRTEM image taken on the tube wall. Reproduced from [85] with permission from American Chemical Society, 2020. (**B**) SEM image of magnetite nanorods described in [71]. Reproduced from [71] with permission from Elsevier, 2020. (**C**) Scheme of the two-step synthesis of magnetite nanorods. Adapted from [88] with permission from Royal Society of Chemistry, 2020.

Nanorods have been commonly obtained through hydrothermal reactions, which provides an economical and convenient route for the synthesis of crystals with compositional and morphological control, without requiring extremely high temperatures and sophisticated processing [75,78]. On the other hand, nanowires and nanotubes synthesis is commonly accomplished through template-assisted strategies. Opposite to other strategies, a reproducible method for the synthesis of homogeneous magnetite nanotubes (Figure 1A) was developed by Liu et al. [85], which consisted on the synthesis of MgO nanowires grown on Si/SiO_2_ substrates, pulsed laser deposition of Fe_3_O_4_ onto the nanowires and etching of the MgO inner cores with (NH_4_)_2_SO_4_. Yet, magnetic nanorods have been of higher interest as theranostic agents. Through the hydrothermal method, Wan et al. [71], using ethylenediamine as a shaping ligand, were able to synthesise uniform nanorods (Figure 1B), which were achieved later in a greener synthesis strategy by Ding et al. [70] without use of benzene. A tuneable strategy for the synthesis of magnetite nanorods (Figure 1C) has also been developed by Mohapatra et al. [88], which consists on the synthesis of PEI coated FeOOH nanorods and its reduction with oleylamine. The control of size between 25 nm and 70 nm is attained by varying PEI concentration, which adsorbs on the plane {200} of the nanorods [89,90]. The nanorods are then reduced to magnetite by oleylamine that acts as a reducing agent (electron donor) at 200 °C, besides acting as surfactant and solvent [88,91].

Nanorods of CoFe_2_O_4_ are commonly obtained through a shaping ligand-assisted synthesis using oleic acid or CTAB as a surfactant [77,78,92]. Cobalt ferrite nanoparticles have remarkable properties, such as tuneable coercivity, large anisotropy and moderate saturation magnetization [77]. Yet, their application in the biomedical field is restricted owing to cobalt toxicity. Other ferrites, such as MnFe_2_O_4_, CaFe_2_O_4_ and MgFe_2_O_4_, have advantageous properties for biomedical applications; although, strategies for the synthesis of nanorods of these materials are lacking.

### 2.2. Nano- Films, Sheets and Plates

Two dimensional materials consist of plate-like magnetic nanoparticles, which magnetization in soft-magnetic materials lies parallel to the basal plane, requiring a strong magnetic field to align the magnetization out of the plane [23].

Thin films of magnetite have been commonly obtained through deposition techniques such as molecular beam epitaxy and pulsed-laser deposition [93]. Recently, Zukova et al. [93] were able to grow magnetite films using pulsed injection metallorganic chemical vapour deposition (PI MOCVD) on Al_2_O_3_ (0001) and MgO (001) substrates, which displayed a preferred (111) crystalline orientation. The authors denoted that the magnetic properties could be tuned through application of a 1T magnetic field during the film growth and also upon cooling. Another strategy to obtain magnetic nanofilms is the layer-by-layer method. For example, Grigoriev et al. [94,95] were able to synthesize polyelectrolyte/magnetite nanoparticle multilayer films by the layer-by-layer self-assembly technique and evaluated the dependence of the refractive index on the increase of layers.

**Figure 2:**
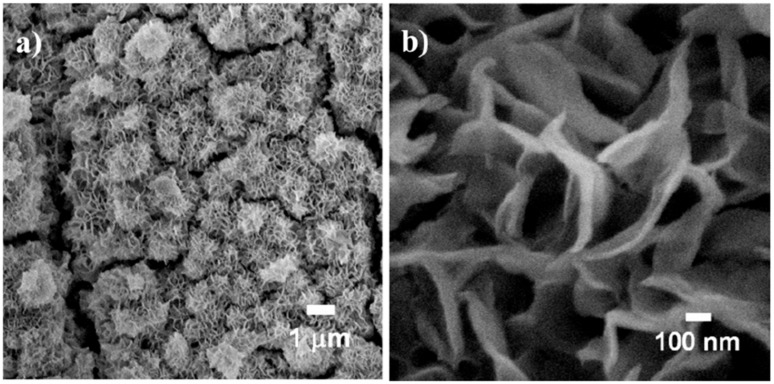
SEM image at different magnifications of magnetite nanosheets. Adapted from [100] with permission from American Chemical Society, 2020.

Other bidimensional shapes, such as nanosheets or nanoplates, have attracted more attention than nanofilms. Different strategies have already been used to obtain magnetite nanosheets, including hydrothermal synthesis [96], or soft-template assisted synthesis [97], affording nanosheets with good crystallinity and magnetic properties. A simple strategy was developed by Wang et al. [98], which consists of microwave-assisted coprecipitation of ferrous sulphate in a mixture of ethylene glycol and water, affording nanosheets with good crystallinity and saturation magnetization around 30 emu/g. Zhuang et al. [99] were able to synthesize magnetite nanosheets through a solvothermal method using diethylene glycol, which, through the inhibition of the crystal plane (111), favours the formation of magnetite sheet-like crystals. Furthermore, the authors demonstrated that the nanoparticles self-assemble into chain-like structures along the direction of the external magnetic field, known as photonic crystals, which display a magnetochromatic property. A scale-up of the synthesis of good quality and homogeneous nanosheets (Figure 2) has been developed by Chin et al. [100], which consisted in the immersion of Fe substrates into a 70 °C solution of HCl and KCl under an O_2_ rich environment. Strategies have also been developed for the synthesis of nanosheets based on other ferrites, mainly CoFe_2_O_4_ through template assisted [101,102,103], and thermal decomposition [104].

**Figure 3:**
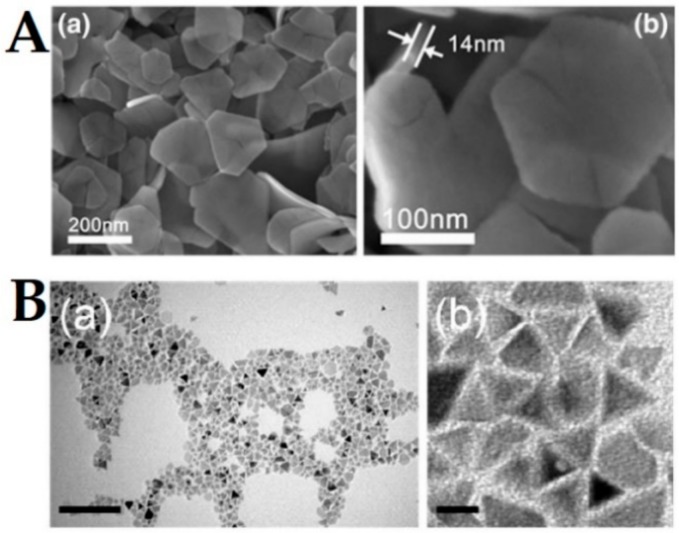
(**A**) SEM images of hexagonal-shaped magnetite nanoplates at different magnifications. Reproduced from [109] published in Open Access by Springer. (**B**) TEM images of triangle-shaped magnetite ultra-thin nanoplates at different magnifications. Adapted from [110] with permission from Royal Society of Chemistry, 2020.

Magnetite nanoplates synthesis have also been reported through a hydrothermal route [105,106], with a saturation magnetization above 60 emu/g. An alternative to hydrothermal route has been proposed by Chen et al. [107], through the use of coprecipitation assisted with ultrasound irradiation, which does not require protection from oxygen and high temperatures, producing nanoplates with thickness ranging from 10 nm to 20 nm and lateral size between 50 nm and 90 nm. Hexagonal nanoplates have been obtained by Zhut et al. [108] through a hydrothermal synthesis using β-cyclodextrin and urea as, respectively, reducing agent and growth modifier. The nanoplates exhibit an average diameter of 400 nm and thickness of 40–80 nm, attaining a saturation magnetization close to 80 emu/g. Ma et al. [109] also synthesized hexagonal nanoplates with maximum magnetization above 70 emu/g through the Schikorr reaction in a hydrothermal route (Figure 3A), which is the formation of iron oxide and molecular hydrogen through oxidation of iron(II) hydroxide under anaerobic conditions. Recently, Xu et al. [110] were able to synthesize ultra-thin triangular magnetite nanoplates through a seed-mediated synthesis, where the seeds were obtained through the thermal decomposition of ferric oleate complex at 310 °C, and a growth-step (replenished with monomers) was carried out at a temperature (240 °C) lower than the required for nucleation (310 °C), but higher than the thermal decomposition of the complex (230 °C).

Recently, the synthesis of Co_1-x_Cu_x_Fe_2_O_4_ has been achieved through a two-step synthesis, starting with coprecipitation of the metal salts in the right ratio, which dried powder was subjected to thermal decomposition in air at 400 °C in a tubular furnace [111]. Mn_x_Fe_1-x_O nanoplates has also been recently reported, which are of biomedical interest owing to the resistance to oxidation in air compared to MnO and FeO, making them good candidates for magnetic contrast agents [112]. The synthesis was achieved through thermal decomposition of acetonate precursors under N_2_ atmosphere, affording nanoparticles that reach a saturation magnetization of 30 emu/g. The synthesis of Ni_1-x_Mn_x_Fe_2_O_4_ thin cubic nanoplates has also been obtained through coprecipitation of the metal salts in the right ratio and assisted with oleic acid as surfactant, to avert agglomeration during the reaction [113,114].

### 2.3. Nanocubes and Flowers

**Figure 4:**
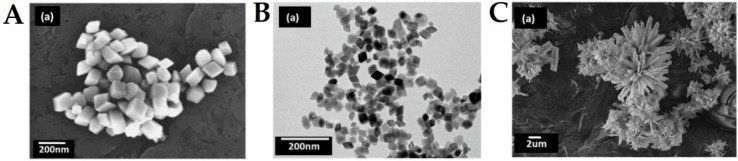
(**A**) SEM image of distorted cubes. (**B**) TEM image of nanocubes. (**C**) SEM image of self-oriented flowers. Reproduced from [115] with permission from Springer Nature, 2020.

Despite the already described advances in the synthesis of colloidal iron oxide magnetic materials, efforts are still ongoing to attain facile and sustainable synthetic protocols for controlling the shape of nanoparticles. A protocol that allows control of nanoparticles was developed by Sayed and co-worker [115], in which through a microwave assisted method and by changing only the iron precursor, different shapes could be obtained such as distorted cubes, nanocubes and flowers (Figure 4).

A thermal decomposition method to synthesize magnetite flowers was developed by Guo et al. [116] using FeCl_3_.6H_2_O, urea and tetrabutylammonium bromide dissolved in ethylene glycol, which afforded particles with an average diameter of 1–3 µm and a saturation magnetization up to 80 emu/g. Later, Wang et al. [117] followed the reaction mechanism through electron microscopy, which proceeds as nucleation, aggregation and Ostwald ripening, and finally the oriented growth was also accompanied by Ostwald ripening. Flower-like particles have also been obtained through solvothermal routes. Li et al. [118] synthesized flower microspheres through solvothermal reaction with urea in ethylene glycol (200 °C, 4 h), which afforded particles that reach a magnetization in saturation up to 80 emu/g. A different particle architecture was developed by Wang et al. [119] through the solvothermal route, using CTAB and sodium acetate in ethylene glycol (200 °C, 24 h), that afforded hollow microspheres with a M_s_ of 80 emu/g. Other flower-like architecture is exhibited by the multicore particles, in which the interaction between the cores leads to a large magnetic moment and weak remanence magnetization [120]. Gavilán et al. [120] developed different methods to synthesize multi-core nanoparticles through the use of polyols as reducing agents, obtaining nanoflowers as small as 50 nm and M_s_ value of 100 emu/g. Through the use of succinic acid, urea and FeCl_3_.6H_2_O in a solvothermal route, Cheng et al. [121] were also able to synthesize flower-like multi-core nanoparticles with *ca.* 300 nm, while the use of 1,2-propylene glycol as solvent afforded nanoparticle clusters with *ca.* 50 nm. Recently, Shubitidze et al. [122] reported a coprecipitation method using dextran and NaNO_3_ that afforded nanoflowers with sizes of 20 nm to 40 nm.

**Figure 5:**
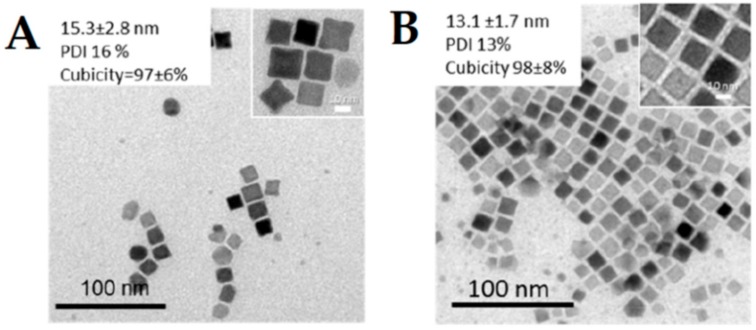
TEM image of (**A**) CoFe_2_O_4_ and (**B**) MnFe_2_O_4_ nanocubes. Adapted from [126] with permission from American Chemical Society 2020.

Anisotropic nanoparticles have been sought as a solution to improve T_2_ contrasting properties as the relaxivity can be enhanced by increasing the effective radius of the magnetic core, which is majorly morphologic dependent [123]. For example, Zhao et al. [100] synthesized octapods (Figure 5A) through thermal decomposition of iron oleate in the presence of NaCl. This strategy afforded nanoparticles with an average length of 30 nm, which displayed an ultrahigh transverse relaxivity value of 679.3 30 mM^−1^ s^−1^. Common methods for the synthesis of nanocubes include thermal decomposition of iron(II) oleate or iron(III) acetylacetonate complexes [124,125]. Recently, Muro-Cruces et al. [126] synthesized cubic MFe_2_O_4_ (M = Fe, Co, Mn) (Figure 5A,B) and Mn_3_O_4_ through the thermal decomposition of the right ratio of metal acetylacetonates in the presence of sodium oleate and oleic acid, in a mixture of 1-octadecene, dibenzyl ether and 1-tetradecene. Through this pathway, the authors were able to control the size variation in 1–2 nm and obtain monodisperse nanoparticles with high crystallinity, which is a critical control requirement to develop new materials for decreasing nuclear magnetic resonance relaxation time T_2_.

## 3. Biomedical Applications of Anisotropic Iron Oxide Nanoparticles

The use of iron oxide nanoparticles (IONs) in cancer therapy and diagnosis (theranostics) has been of major interest regarding their unique physicochemical properties, namely, their superparamagnetic behavior, high magnetization and chemical stability [127,128,129]. The already reported influence of shape in the physicochemical properties and magnetic behavior of IONs [130,131,132,133] was a starting point to further research on the impact of shape anisotropy in biological behavior and imaging properties. Next sections are focused on recent developments in magnetic drug delivery, magnetic hyperthermia and magnetic resonance imaging (MRI).

### 3.1. Magnetic Drug Delivery

IONs can accumulate in tumor sites by passive or active targeting. Passive targeting occurs when nanoparticles can extravasate from the bloodstream and enter in tumor cells through the enhanced permeability and retention (EPR) effect [134,135]. On the other hand, active targeting with an applied magnetic field takes advantage on the responsiveness of magnetic nanoparticles towards a magnetic field [136,137]. IONs can also be coated with synthetic and natural polymers [130,138,139], surfactants and fatty acids [31,140] and functionalized with targeting ligands [130,141], which allows the use of these nanoparticles as drug delivery systems with improved selectivity and pharmacokinetics [142,143].

**Figure 6:**
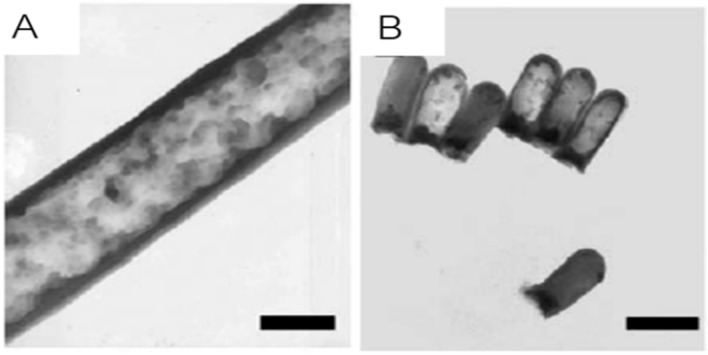
TEM images of 200 nm (**A**) and 60 nm (**B**) nanotubes with magnetite. Scale bars, 200 nm. Adapted from [147] with permission from Elsevier, 2020.

The superparamagnetic properties demand that nanoparticles have small sizes, preferably below ~20 nm [4]. However, at this size, magnetic moments of nanoparticles are small, so magnetic response can be compromised. Self-assembly of individual nanoparticles into nanoclusters is one possible strategy to overcome this problem. Kralj et al. [144] developed nanochains and nanobundles from nanoclusters of maghemite (γ-Fe_2_O_3_) with preserved superparamagnetism, zero coercivity and good colloidal stability. Other elongated structures, like nanotubes or nanorods, are also being investigated for drug delivery. Iron oxide nanorods have attracted attention due to their superparamagnetic behavior [145] and capacity of intracellular delivery with controlled-release profile and biocompatibility [146]. Nanotubes have the advantage of enabling the loading of large amounts of bioactive compounds in their inner voids, while the outer surface can be coated or functionalized with targeting ligands (Figure 6A,B) [147].

**Figure 7:**
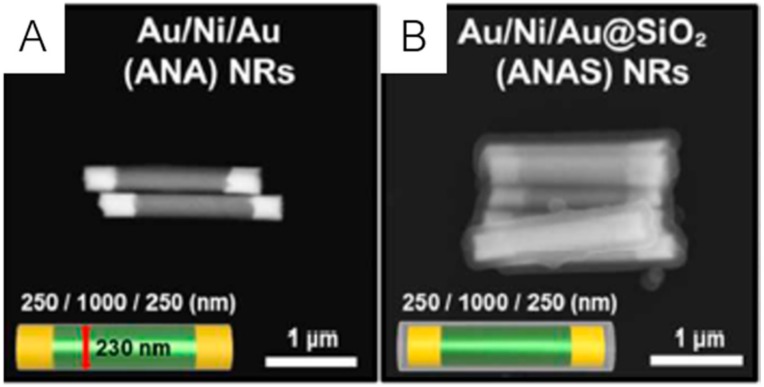
FE-SEM images of Au/Ni/Au nanorods (**A**) and silica-coated Au/Ni/Au nanorods (**B**) and respective schematic representations. Adapted from [65] with permission from Royal Society of Chemistry, 2020.

Magnetic nanotubes combine the magnetic and tubular properties allowing a differential functionalization of the inner and outer surfaces, which shows the potential of these nanostructures for magnetically assisted drug delivery and bioimaging [148]. A study regarding the use of iron oxide nanotubes as carriers for insoluble anticancer drugs showed that hematite nanorods were in a higher extent and more quickly internalized than nanospheres in human carcinoma cells [149]. After achieving this conclusion, the researchers developed PEGylated maghemite nanorods loaded with paclitaxel (PTX-PMNTs) and demonstrated that these nanocarriers have important properties for a successful magnetic drug delivery such as higher drug loading capacity, increased carcinoma uptake in the presence of a magnetic field and a pH-activated release profile. In 2014, Yu et al. [150] synthesized iron oxide porous nanorods functionalized with folic acid for targeted delivery of another low water-soluble anticancer drug, doxorubicin. In this study, the researchers demonstrated that the presence of folic acid on the surface of nanorods increased cellular uptake of nanorods and cytotoxicity of doxorubicin in HeLa cells, because of the specific binding between folic acid and folate receptors, thus showing the potential of these nanocarriers for targeted drug delivery in tumor cells. More recently, an innovative nanosystem was developed by Kwak et al. [65], being composed of Au blocks (plasmonically-active domain) and Ni blocks (magnetically-active domain), with a silica-coated surface for drug loading (Figure 7A,B).

**Figure 8:**
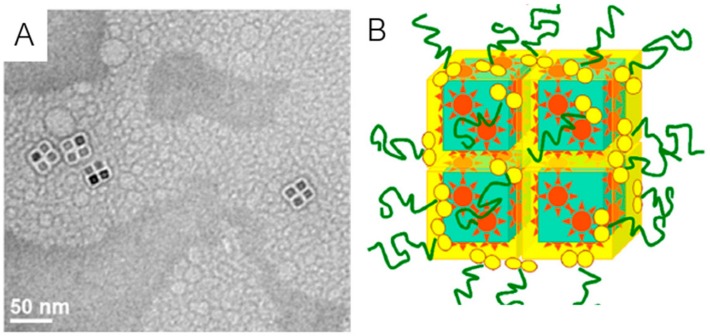
TEM image (**A**) and schematic representation (**B**) of Rubik-like paclitaxel-loaded magnetic nanoassemblies. Adapted from [151] with permission from Elsevier, 2020.

A rotating magnetic field will induce fluctuations of magnetic-responsive parts and consequently produce periodic fluctuations in optical measurements that can be converted by Fourier transform in a dominant frequency peak. The release rate of loaded drug can be controlled by changing the speed of the applied magnetic field, which in turn, is monitored by frequency peak shifting. In this study, doxorubicin was loaded in these nanorods and its release rate was higher when the speed of the rotating magnetic field increased, confirming the controlled release using magnetic modulation. Besides that, a linear decrease in cell viability was shown in HeLa cells as a function of magnetic field speed and release time. In 2013, Xiong et al. [151] produced Rubik-like magnetic nanoassemblies (MNAs) composed of four oleic acid-capped iron oxide nanocubes (Fe_3_O_4_@OA NCs) with a shell of dioleate-modified polyethylene glycol (OA2-PEG) (Figure 8A,B). Here, the researchers investigated the antitumor effect of paclitaxel-loaded MNAs in vitro using B16F10 culture cells and in vivo in B16F10 melanoma-bearing mice and demonstrated higher antitumor activity of these nanosystems, compared with the same amount of free paclitaxel (also known as taxol), as well as an enhanced intracellular delivery in the presence of a magnetic field. The pharmacokinetics study allowed concluding that the incorporation of paclitaxel in MNAs increased the amount and retention time of the drug in plasma, also an important parameter to be considered in drug delivery.

### 3.2. Magnetic Hyperthermia Therapy

Cancer cells can be killed when exposed to high temperatures between 41 and 47 °C in a process known as hyperthermia [152]. Magnetic hyperthermia can be induced when magnetic nanoparticles are exposed to an alternating magnetic field (AMF) and produce heat due to Néel and Brownian relaxations of the rotating magnetic moments [152,153]. Metallic nanoparticles (Fe, Ag, Ni, Gd, TiO_2_) have the highest saturation magnetization, but their inherent toxicity and chemical instability make them not suitable for biomedical applications. Thus, the most used nanoparticles for hyperthermia applications are iron oxide nanoparticles that show low toxicity, facile fabrication and advantageous physicochemical properties [153,154]. This treatment can be combined with specific delivery of therapeutic drugs into the tumor cells, in order to increase the therapeutic effect and reduce the needed dosage and side effects [152,153,154].

The heating power of magnetic nanoparticles strongly depends on extrinsic and intrinsic properties, namely the external magnetic field amplitude, the saturation magnetization and the anisotropy energy, which in turn, depends on shape of nanoparticles, so a suitable design of these nanoparticles is an important step for enhanced hyperthermia efficiency [155]. The increase of heating efficiency allows the use of a lower dose of nanoparticles, preventing this way undesirable accumulation and cytotoxicity.

**Figure 9:**
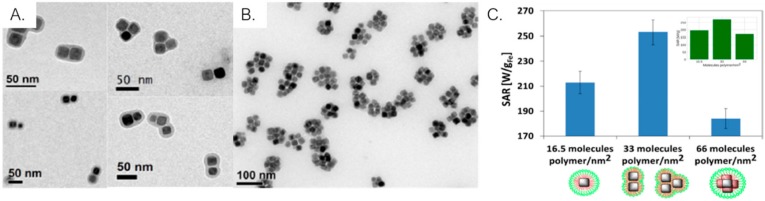
TEM images of dimer and trimer (**A**) and centrosymmetric (with more than 4 nanocubes) (**B**) iron oxide nanoclusters; (**C**) SAR values of the different nanoassemblies shows that dimer and trimer structures have the highest SAR values. Adapted from [158] with permission from American Chemical Society 2020.

Nanocubes have been extensively studied for hyperthermia applications. Guardia et al. [156] reported significant SAR values of nanocubes with 19 nm of edge length, at frequencies and magnetic field amplitudes considered safe for clinical use (a SAR value of 875 W/g at 320 kHz and 10 kA.m^−1^), as well as high hyperthermia efficiency, promoting 50% of cancer cell mortality in a temperature range of 40 to 45 °C, at 110 kHz and 20 kA.m^−1^. Thermo-sensitive coated nanocubes have the ability of combining hyperthermia with chemotherapy by creating a synergistic therapeutic effect through heat triggered drug release, while maintaining their good magnetic properties and high SAR values [157]. The coating of magnetic nanoclusters seems to be a suitable strategy to create colloidally-stable solutions of nanoparticles aggregates taking advantage of magnetic and heating properties of different geometries. Niculaes et al. [158] prepared and compared oleic acid-coated iron oxide nanocubes with different geometries – single nanocubes, dimer and trimer assemblies, and centrosymmetric structures (with more than 4 nanocubes) – in terms of their magnetic properties and SAR values (Figure 9A–C). The SAR value of dimer and trimer structures was higher (253 W/g) than the one obtained for individual nanocubes (213 W/g) and a significantly decrease was observed for centrosymmetric assemblies (184 W/g). Dimer and trimer nanoclusters also had the highest saturation magnetization (M_S_) values. However, it was demonstrated that not Ms but magnetic dipolar effect is the major factor for the enhancement of SAR values (and not the M_S_ values).

Geng et al. [159] demonstrated that iron oxide nanorods show enhanced specific absorption rates (SAR) (1072 W/g at 33 kA.m^−1^) comparing with their spherical counterparts (262 W/g). Other comparative study regarding the magnetic properties and heating efficiency of magnetite nanorods and spherical nanoparticles allowed demonstrating that the samples of nanorods possess higher Ms values than the ones with spherical nanoparticles of the same volume [160]. Then, AC (alternating current) hyperthermia experiments showed that the increase of the aspect ratio (for a limited concentration of nanoparticles, 3 mg/mL) and alignment of nanorods in the direction of the AC field are key factors for an enhanced heating efficiency. Additionally, the researchers obtained higher SAR values for magnetite nanorods (862 W/g at 800 Oe) when compared to nanocubes and nanospheres (314 W/g and 140 W/g, respectively).

**Figure 10:**
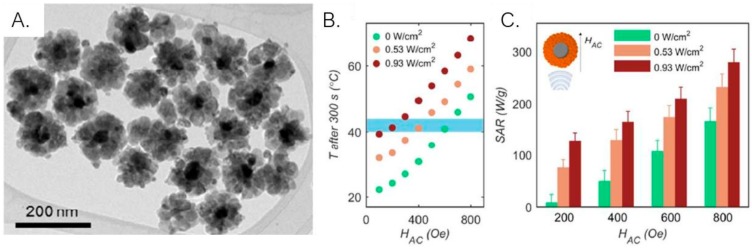
**(A)** TEM image of Ag/Fe_3_O_4_ nanoflowers; (**B**) Temperature reached after 300 s of heating for different ac field intensities. The therapeutic temperature window (blue zone) is reached for lower magnetic field intensities when a laser is applied; (**C**) SAR values of Ag/Fe_3_O_4_ nanoflowers as a function of AC field intensity (HAC) for different laser power densities. SAR values are the highest when a laser is simultaneously applied. Adapted from [162] with permission from American Chemical Society, 2020.

Not only nanorods and nanocubes are promising enhancers of heating efficiency. Magnetite nanodiscs were also investigated and, again, higher SAR values (2 times higher) for nanodiscs were obtained in comparison with their spherical counterparts [161]. More important, it was demonstrated that the high SAR value for nanodiscs in an aqueous solution (4.66 kW/g) is due to their parallel orientation to the applied AC field and can be maintained in a range of orientations of 60° to 90°. Ag/Fe_3_O_4_ core-shell nanoflowers were used in combined magnetic hyperthermia and photothermal therapy, taking advantage of both plasmonic and magnetic properties and allowing an increase of more than one order of magnitude of SAR values when a laser is simultaneously applied (Figure 10A–C) [162].

As mentioned before, nanotubes have high M_S_ and increased surface area that allows higher drug content due to their hollow morphology. The possibility of enhancing the therapeutic efficiency of a drug is now of major importance, justifying the development of multifunctional nanoparticles capable of a synergistic effect between efficient heating and drug delivery. Following this, Das et al. [163] prepared highly crystalline Fe_3_O_4_ nanotubes and measured the SAR values, in water and in 2% agar, for randomly dispersed and magnetically aligned nanotubes. A SAR value of 360 W/g for an AC magnetic field of 800 Oe was obtained for randomly dispersed nanotubes in water. For applied fields of 600 and 800 Oe, nanotubes aligned to the magnetic field had improvements in SAR of 65% and 80%, respectively, comparing to randomly dispersed nanotubes. This, again, confirms the importance of the alignment of anisotropic nanoparticles to the applied magnetic field in order to enhance the heating efficiency.

### 3.3. Magnetic Resonance Imaging (MRI)

Iron oxide nanoparticles have been extensively exploited as enhanced contrast agents in magnetic resonance imaging (MRI). The main advantages of MRI are high spatial resolution, soft tissue contrast and, most important, the possibility to early detect the presence of tumors, increasing therapy success [164]. Thus, the conception of new strategies that can optimize this technique is of major relevance. Superparamagnetic iron oxide nanoparticles (SPIONs) are strong candidates to be used as contrast agents due to their biocompatibility and ability to increase contrast enhancement [165]. A contrast in MRI image depends on nuclear relaxation times of the tissue protons, which can be longitudinal (T_1_) or transversal (T_2_) [166,167,168]. Positive contrast agents (T_1_) decrease longitudinal relaxation time, resulting in a brighter image, while negative contrast agents (T_2_) decrease transversal longitudinal relaxation time, causing a darkening in MRI image [166,167,168]. The behavior of a contrast agent essentially depends on longitudinal and transversal relaxivity, r_1_ and r_2_, respectively, indicating if it is more susceptible to be positive (T_1_) or negative (T_2_) [166,167,168]. Commonly, SPIONs are used for darkening T_2_-weighted images, but it was already reported that these nanoparticles are also capable to provide positive contrast enhancement, overpassing the toxicity of the usual gadolinium chelates contrast agents [169,170,171].

**Figure 11:**
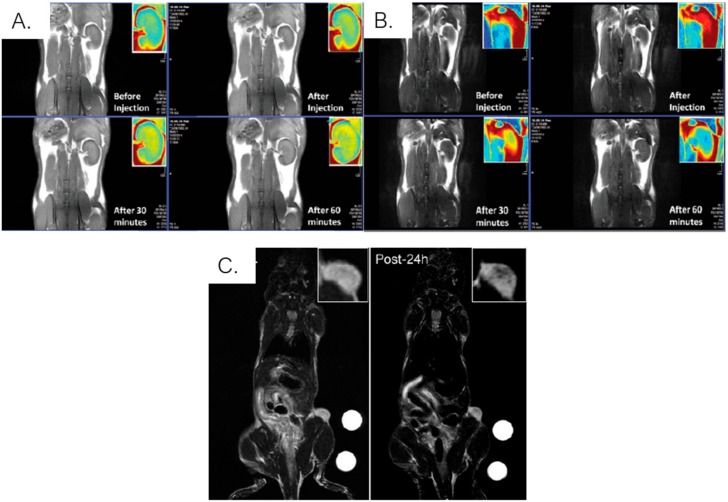
T_1_- (**A**) and T_2_-weighted (**B**) in vivo MR images before injection, immediately after and after 30 and 60 minutes of silica coated nanocubes. The contrast enhancement is clearly observed in the coronal images of the kidney. Adapted from [124] with permission from Royal Society of Chemistry 2020. (**C**) T2-weighted in vivo MR images before (left) and 24 h after (right) injection of BSA-coated nanocubes into mice bearing U87-MG tumor cells. The darkening of the tumor mass is clearly visible after the injection of nanoparticles. Adapted from [175] with permission from MDPI 2020.

The beforementioned studies regarding the use of iron oxide nanoparticles in drug delivery and hyperthermia treatment evidenced that anisotropy of nanoparticles is a key factor for improving its properties. This way, researchers started to investigate the morphology dependence of nanoparticles in the enhancement of magnetic relaxivity in MRI [172,173]. Lee et al. [174] demonstrated that 22 nm-sized nanocubes have a higher r_2_ value (761 s^−1^ mM^−1^) compared with nanocubes with larger sizes and clear attenuation of the MRI signal in vivo, which opens the possibility of using nanoparticles with smaller sizes and high colloidal stability as T_2_ contrast agents. Sharma et al. [124] synthesized even smaller magnetite nanocubes (9.7 nm) with potential of simultaneous contrast enhancement of T_1_ and T_2_-weighted MRI, acting as dual contrast agents (Figure 11A,B). Later on, Cho et al. [175] prepared BSA-coated assembled iron oxide nanocubes with high r_2_ relaxivity (~500 s^−1^ mM^−1^). These nanoparticles, with an average size of 100 nm, were injected into mice bearing U87-MG tumor cells and a clear darkening of tumor mass was detected, confirming their accumulation (Figure 11C). Moreover, the tumor growth was successfully reduced with magnetic hyperthermia, which demonstrates the potential of these nanoparticles in combined diagnostics and therapy. Zhao et al. [123] synthesized iron oxide octapods with an edge length of 30 nm that exhibited a stronger contrast enhancement of T_2_-weighted images and a much higher r_2_ value (679 s^−1^ mM^−1^) than spherical iron oxide nanoparticles with similar geometric volume. Furthermore, these nanoparticles produced higher contrast in T_2_-weighted MR imaging of a hepatic carcinoma than the spherical ones, indicating that iron oxide octapods are efficient as T_2_ contrast agents. These results demonstrate that anisotropic shapes promote the increase of the effective radii of nanoparticles enhancing their performance in T_2_-weighted MRI. While the r_2_ values are influenced by not only the effective radii but also the saturation magnetization, r_1_ values depend mostly on metal exposure at the surface of nanoparticles that promotes proton coordination and chemical exchange [176,177]. This was emphasized by Zhou et al. [178] with the preparation of several anisotropic iron oxide nanostructures, namely Fe_3_O_4_{111} facet exposed plates, truncated octahedrons and tetrahedrons, and Fe_3_O_4_{100} facet exposed cubes, concaves, multibranches and assembled structures. In this study, the r_1_ and r_2_ values were obtained and compared for all nanostructures and it was demonstrated that Fe_3_O_4_{111} exposed cubes, octahedrons, tetrahedrons and Fe_3_O_4_{100} exposed cubes (with sizes of both 15 and 21 nm) have a greater T_1_ relaxation shortening effect than spherical nanoparticles, because of the metal-rich surfaces. The concaves and multibranches had lower r_1_ values similar to the one for spherical nanoparticles. The highest r1 and r_2_ values were obtained for 21 nm sized cubes, possibly due to spin-canting effect, in the case of T_1_ relaxation, and higher M_s_ and relatively large effective radii, in the case of T_2_ relaxation, when comparing with the remaining nanoparticles.

Among the elongated nanoparticles, nanorods were mentioned before as efficient systems for hyperthermia treatment because of their high M_S_ values. Knowing that higher magnetization leads to a shortening effect of T_2_ relaxation time, it can be expected that nanorods are good T_2_ contrast agents. In fact, Mohapatra et al. [88] reported a high r_2_ value (608 s^−1^ mM^−1^) for magnetite nanorods of length 70 nm coated with polyethyleneimine (PEI). These nanorods were able to induce a stronger local magnetic field over a larger volume comparing with spherical nanoparticles with equivalent volume, due to their larger surface area, resulting in a faster dephasing of nearby protons and consequently a higher r_2_ value is obtained. Later, the same group [179] prepared silica-coated magnetite nanorods (Fe_3_O_4_@SiO_2_) with a length of ~520 nm and an r_2_ value of 192 s^−1^ mM^−1^. The nanorods promoted an r_2_ darkening effect, exhibited high loading efficiency (65%) of doxorubicin and a pH-stimulated release of the drug. These results indicate the potential of nanorods for combined therapy and diagnosis. The treatment and imaging of colon cancer in mice was investigated by Dehvari et al. [180], who developed pluronic-conjugated superparamagnetic nanorods loaded with paclitaxel. Here, tumor growth was suppressed, and in vivo localization and visualization of the tumor was possible due to the darkening effect promoted by the nanorods in T_2_-weighted MR images. More recently, elongated magnetite nanoparticles were synthesized by an environmentally friendly hydrothermal method, and it was demonstrated that the presence of larger pores resulted in the highest M_S_ value (97 emu g^−1^) and transversal relaxivity (r_2_ = 343 s^−1^ mM^−1^) [63]. It is worth to note that the abovementioned iron oxide nanoparticles have higher transversal relaxivity values than the ones obtained for clinically approved T_2_ contrast agents, such as Ferumoxide (Endorem^®^ or Feridex^®^) and Ferrixan (Resovist^®^) with r_2_ values of ~98 s^−1^ mM^−1^ [181] and ~150 s^−1^ mM^−1^ [182], respectively. More efficiency is now being achieved in anisotropic nanoparticles for MRI applications and further research on their effects is needed, namely in vitro and in vivo studies, in order to be clinically approved.

## 4. Cytotoxicity of Iron Oxide Nanoparticles

Besides the development of new strategies of synthesis and production of nanoparticles with different structural characteristics, the interaction of nanoparticles with biological systems is an important factor to be considered for long-term clinical use. The uptake and accumulation of iron oxide nanoparticles in tumor cells will depend on their size, shape, surface charge and coating. It is desirable that a great number of nanoparticles reaches the tumor environment without causing any harm to healthy cells when circulating inside the body. This way, controlled size and shape of iron oxide nanoparticles are fundamental parameters to be considered in their design for biomedical applications. The range of sizes from 10 nm to 100 nm has been reported as the ideal. Nanoparticles with sizes below 10 nm are easily cleared by kidneys, while nanoparticles with sizes larger than 100 nm are phagocytized by macrophages and accumulate in the liver and spleen [183]. Cytotoxicity promoted by iron oxide nanoparticles can result from different mechanisms, such as reactive oxygen species (ROS) production, oxidative stress, membrane damage, genotoxicity and inflammatory responses [184,185]. ROS production is mainly due to the large surface area of iron oxide nanoparticles for generation of free radicals and leaching of iron molecules from the surface by enzymatic degradation. This will lead to oxidative stress and possibly causing cell death by apoptosis or necrosis. The most common way of overcoming this problem and achieve colloidal stability is the surface coating of nanoparticles with different polymers such as dextran, polyethylene glycol (PEG) and liposomes [186].

However, some coatings can cause appreciable cytotoxicity. For example, Feng et al. [187] demonstrated *in vivo* dose-dependent lethal toxicity of 10 nm poly(ethylenimine)-coated iron oxide nanoparticles. Also, Ying et al. [188] concluded that, in a 24 h assay, iron oxide nanoparticles coated with carboxyl groups have higher toxicity than those with amine groups in A3 human T lymphocytes. Jarockyte et al. [189] prepared magnetite nanoparticles with a hydrodynamic size of about 50 nm and studied the accumulation and toxicity of these nanoparticles in mouse embryonic fibroblasts. After 48 h, nanoparticles were mainly accumulated in the perinuclear region. Then, cell viability was tested for 3 h, 24 h and 48 h, with incubation of 32.5 ng/mL and 65 ng/mL of nanoparticles. After 3 h and 24 h, no cytotoxicity was observed for both concentrations and, after 48 h, a dose-dependent decrease in cell viability is observed.

Not only size but also the morphology of nanoparticles can influence cytotoxicity [190,191]. *In vitro*, shape will mostly influence the nanoparticle-cell membrane interaction, which in turn will influence cellular uptake. Rod-shaped nanoparticles seem to have a more favorable cellular uptake when compared with spherical nanoparticles, due to the larger area of contact between the cell and the nanoparticle, so it is expectable that nanoparticles with this shape will accumulate in higher extent in tumor cells. On the other hand, a higher accumulation of these nanoparticles can lead to cell necrosis of non-tumorigenic cells, as demonstrated by Lee et al. [192]. In this study, rod-shaped iron oxide nanoparticles were found to be more cytotoxic to mouse macrophage cells than the spherical counterparts. On the other hand, Hu et al. [193] showed that iron oxide nanocubes produced low cytotoxicity up to 0.5 mg Fe mL^−1^ in a mice monocyte macrophage cell line.

*In vitro* and *in vivo* studies show that cytotoxicity is size-, shape- and dose-dependent, but these parameters differ according to the type of cell or tissue in which nanoparticles are being investigated [194,195,196,197]. So, further investigation on iron oxide nanotoxicity in different types of tissues is needed to achieve a more consistent knowledge on which range of sizes is the most suitable for a certain organ and which concentration is safe to be applied.

## 5. Conclusions and Future Perspectives

Over the past years, many different strategies have been developed for the reproducible synthesis of high-quality monodisperse anisotropic iron oxide-based magnetic nanoparticles, either through physical, chemical or biological methods. Although most of the developed strategies are focused solely in magnetite, methods for the synthesis of cobalt, manganese or nickel-doped ferrites have started to be a target of research. Remarkably, the number of reported syntheses of anisotropic shape nanoparticles has been continuously increasing, which is expected to improve further owing to the progress of scientific theories ruling the shape control. Yet, scaling remains a problem to be addressed and an evaluation of the therapeutic gains over the cost of production needs to be evaluated in future works, so to facilitate shape anisotropic synthesis at industrial scale with the desired properties.

Considering the nanoparticles shapes discussed in this article, the synthesis of elongate-shaped nanoparticles has been of major interest for drug delivery applications, owing to the superparamagnetic behaviour, the control over drug-release profiles and biocompatibility. Furthermore, elongated nanoparticles are able to induce a stronger local magnetic field over a larger volume than spherical nanoparticles with equivalent volume, making them highly important as T_2_ contrast agents. Particularly, nanotubes enable the possibility of differential functionalization of the inner and outer surfaces. Thus, the inner voids can be optimized for the loading of bioactive compounds, while the surface might be coated or functionalized with targeting ligands.

The high saturation magnetization of shape anisotropic nanoparticles allows the use of a lower dose of nanoparticles, averting undesirable adverse and side effects. Particularly, nanorods and nanocubes have shown promising hyperthermia results for cancer therapy. However, challenges still remain at the chemical composition level, as magnetite is prone to the generation of reactive oxygen species. Such can be overcome through investigation of other chemical compositions, such as ferrites doped with calcium or magnesium, or surface passivation with an inert material.

Hereby, future works should be focused on the facile fabrication of low toxicity anisotropic nanoparticles with other ferrite chemical composition, so that the physicochemical properties can be optimized and reduce the needed dosage of nanoparticles but also of the therapeutic drugs, and their associated side effects. Also, the development of nanoparticles that combine a plasmonically-active domain are of high interest, due to the possibility of taking advantage of both plasmonic and magnetic properties, such as the attainment of higher SAR values when a laser is simultaneously applied without requiring high magnetic hyperthermia conditions.

## Figures and Tables

**Table 1:** Common methods used in the synthesis of iron oxide nanoparticles, advantages and disadvantages, and references. Examples of isotropic and anisotropic shape nanoparticles are included.

Method	Advantages	Disadvantages	Ref.	Examples
Laser Ablation Synthesis in Solution (LASiS)	Green synthesis Different structures and composition	Difficult control of particle size and clustering	[27,28]	[29,30]
Chemical vapor deposition (laser and spray pyrolysis)	Easy to prepare Production of small particle size	Expensive equipment Gaseous interferences	[26,31,32,33]	[34,35,36,37]
Co-precipitation	Green Low-cost Scalable Facile Efficient	Difficult to control size Polydispersity Lack of precise stoichiometric phase control	[26,28,31,32,38]	[39,40,41]
Thermal decomposition	Small size particles Control of size and shape Monodisperse	Requires multiple steps Toxic solvents Toxic and expensive precursors Laborious purification Requires surface treatment after synthesis	[31,33]	[42,43]
Hydrothermal (solvothermal)	Green Versatile Control of morphology	Need of autoclave Control of dispersity Slow reaction kinetics	[28,33]	[44,45]
Sol-gel synthesis	Homogeneous Control of shape and length Low-cost High phase purity	Requires post treatment By-products Safety Low efficiency	[28,33]	[46,47]
Sonochemical decomposition	Mild experimental conditions Good crystallinity Versatile	Mechanism not still understood	[26,32,43]	[48,49]
Microemulsion	Monodispersity Simple equipment High control of size and shape Room conditions Small sizes	Removal of surfactants High solvent consumption Low-yield Difficult scale-up	[26,28,31,33]	[50]
Electrochemical synthesis	Control of particle size Simple and fast	Lack of reproducibility	[32]	[51,52]
Biosynthesis	High yield Reproducibility Scalability Low cost Room temperature	Time-consuming Laborious	[31,32]	[53,54]

**Table 2:** Common methods used in the synthesis of elongated iron oxide-based nanoparticles. The size and saturation magnetization, M_s_, values are also included.

Shape	Structure	Method	Size (*d* × *l*, nm)	M_s_ (emu/g)	Ref.
Nanorods	Fe_3_O_4_	Hydrothermal + shaping ligand (EDA)	40–50 × 500–800	72.94	[70]
			25 × 200	71.3	[71]
		Co-precipitation + shaping ligand (PVP)	18.2 × 310.6	28	[72]
		Hydrothermal + sacrificial template (goethite)	~70 × ~500	90	[73]
		Solvothermal + template-assisted (Fe_3_O_4_ microspheres)	7–20 × 120–400	92.3	[74]
	MnFe_2_O_4_	Hydrothermal + sacrificial template (MnO)	25–40 × 300–400	72.45	[75]
	CoFe_2_O_4_	Hydrothermal	19 × 400	73.36	[76]
		Solvothermal (EG)	100–200 *l*	54.93	[77]
		Hydrothermal + shaping ligand (CTAB)	25 × 120	66	[78]
	Co_0.5_Ni_0.5_Fe_2_O_4_	Microemulsion	30–200 *l*	51.1	[79]
	CuFe_2_O_4_	Co-precipitation	120–400 *l*	-	[80]
Nanowires	Fe_3_O_4_	Sol-gel + shaping ligand (EG and P123)	10 *d*; >500 aspect ratio	34.5	[81]
	MnFe_2_O_4_	Hydrothermal	100–300 *d*	45.9	[82]
	CoFe_2_O_4_	Sol-gel + template-assisted	40 *d*	-	[83]
		Template-assisted	8–10 *d*	51.81	[84]
Nanotubes	Fe_3_O_4_	Template-assisted	30 *d*; 7 nm wall thickness	-	[85]
	CoFe_2_O_4_	Co-precipitation + Template-assisted	217 *d*	65	[86]
		Sol-gel + template-assisted	50 *d* × 1000 *l*; 5 nm wall thickness	-	[87]
